# Reference panel-guided super-resolution inference of Hi-C data

**DOI:** 10.1093/bioinformatics/btad266

**Published:** 2023-06-30

**Authors:** Yanlin Zhang, Mathieu Blanchette

**Affiliations:** School of Computer Science, McGill University, Montréal, Québec H3A 0E9, Canada; School of Computer Science, McGill University, Montréal, Québec H3A 0E9, Canada

## Abstract

**Motivation:**

Accurately assessing contacts between DNA fragments inside the nucleus with Hi-C experiment is crucial for understanding the role of 3D genome organization in gene regulation. This challenging task is due in part to the high sequencing depth of Hi-C libraries required to support high-resolution analyses. Most existing Hi-C data are collected with limited sequencing coverage, leading to poor chromatin interaction frequency estimation. Current computational approaches to enhance Hi-C signals focus on the analysis of individual Hi-C datasets of interest, without taking advantage of the facts that (i) several hundred Hi-C contact maps are publicly available and (ii) the vast majority of local spatial organizations are conserved across multiple cell types.

**Results:**

Here, we present RefHiC-SR, an attention-based deep learning framework that uses a reference panel of Hi-C datasets to facilitate the enhancement of Hi-C data resolution of a given study sample. We compare RefHiC-SR against tools that do not use reference samples and find that RefHiC-SR outperforms other programs across different cell types, and sequencing depths. It also enables high-accuracy mapping of structures such as loops and topologically associating domains.

**Availability and implementation:**

https://github.com/BlanchetteLab/RefHiC.

## 1 Introduction

Technologies such as Hi-C (Lieberman-Aiden et al. 2009), and micro-C (Krietenstein et al. 2020) capture spatial contacts between DNA fragments in genomes, enabling the inference of various aspects of 3D genome organization. These approaches have revealed a hierarchical spatial organization of topological structures of the genome inside nuclei and spatial patterns such as topologically associating domains (TADs), loops, and compartments. These structures are of vital importance to gene regulation and are dynamic within cells (Sanborn et al. 2015). Identifying these spatial patterns, especially at high resolution, requires the availability of high-coverage Hi-C sequencing data. The investigation of fine-scale structures would even require ultrahigh-coverage contact maps (Krietenstein et al. 2020).

While Hi-C and its variants remain the most popular approaches to map chromatin contacts on a genome-wide scale, the analysis of the data they produce is challenging, in large part due to the moderate sequencing depth (typically 200–500 Million valid read pairs) compared with the size of the contact frequency matrices that need to be estimated. The majority of TAD and loop annotation tools are primarily optimized for high-coverage data and may not provide satisfactory results when applied to typical low- to medium-coverage data, though tools like Grinch (Lee and Roy 2021) have been proposed to analyze low-coverage data. To close the gap, many efforts have been undertaken to perform *in silico* enhancement of Hi-C contact maps (Zhang et al. 2018; Liu and Wang 2019; Liu et al. 2019; Cameron et al. 2020). Given a low-coverage Hi-C dataset, contact map enhancement tools seek to predict a dense, high-resolution version of the contact map aiming to reproduce the map that would be obtained through very deep coverage sequencing of the same library. Super-resolution enhancement could in theory enable high-resolution analysis of low-coverage Hi-C data, e.g. through the application of third-party analysis tools to enhanced maps.

Most existing contact map enhancement tools are deep learning (DL) approaches and are inspired by super-resolution algorithms in image processing. As a high-resolution (5 kb per bin) contact map for a single human chromosome contains 10 000–50 000 bins, existing applications usually split contact maps into nonoverlapping blocks and enhance each block iteratively. HiCPlus (Zhang et al. 2018) was the first DL-based tool proposed for this type of tasks. It is a convolutional neural network (CNN) that contains one hidden layer and is trained from low- and high-coverage contact map pairs (respectively the input and target values) by minimizing the mean square error (MSE) loss. Later, Liu and Wang (2019) proposed a deeper CNN with residual connections—HiCNN and trained it following the strategy used in HiCPlus. Similar to super-resolution analysis in computer vision, the MSE loss leads both models to produce blurry predictions (Liu et al. 2019). To alleviate the issue of over-smoothness, more recent approaches utilize generative adversarial (GAN) frameworks in model training. For example, HiCGAN (Liu et al. 2019) is a CNN built upon a generator containing five residual blocks and a discriminator containing three residual blocks. The generator is trained to produce enhanced contact maps from downsampled contact maps, and the discriminator is trained to distinguish high-coverage contact maps from enhanced contact maps. Liu et al. trained HiCGAN with GAN loss and used the generator for prediction. DeepHiC (Hong et al. 2019) furthers model performance by introducing additional terms to the loss function (i.e. MSE, perceptual loss, and total variance) into training. In contrast, conventional tools (Zhou et al. 2019; Cameron et al. 2020) usually treat contact map enhancement as imputation. They enhance Hi-C signals by fitting a Markov Random Field or performing random walk on a Hi-C graph.

Although DL models have achieved significant successes in contact map enhancement, there is still room for improvement, particularly in the enhancement of very low-coverage contact maps. First, most existing tools are trained on data containing 250 M valid read pairs [typically a 16-fold downsampled version of a very high-coverage Hi-C dataset produced for human GM12878 cells data by Rao et al. (2014)], and can only be effectively used to enhance contact maps containing 200–300 M valid read pairs. In addition, similar to super-resolution analysis in computer vision, contact map enhancement is an ill-posed problem as a single low-coverage contact map may correspond to multiple potential high-coverage contact maps. While existing tools can infer high-fidelity predictions, these may not necessarily be correct predictions, especially in sparse regions, potentially leading to false positives in downstream annotation tasks.

To address the issue of ill-posedness in single-image super-resolution, computer vision researchers have introduced additional images to assist with the prediction task. For example, some studies have created databases of image patches and used them to improve prediction accuracy (Yan et al. 2020; Xia et al. 2022). In recent years, incorporating external data have become a popular research direction and have been shown to lead to better models with fewer parameters (Borgeaud et al. 2021). Within Hi-C data analysis, our recent approach—RefHiC (Zhang and Blanchette 2022) achieves superior performance in annotating topological structures (loops and TADs) from a study sample while using a reference panel of other Hi-C datasets as complement. In reference-based image super-resolution, the reference database is assumed to contain a diverse set of images, regardless of their relationship to the test image. In 3D genome analysis, the conformation of a small region in one cell type may be observed in another cell types (Zhang and Blanchette 2022). Therefore, to improve the resolution of a small region of a Hi-C contact map, we use contact maps of the same region as a reference.

Here, we introduce RefHiC-SR, a model for enhancing Hi-C contact maps. While RefHiC (Zhang and Blanchette 2022), our model for topological structure annotation, is limited in its ability to learn features from large patches required in a super-resolution task, RefHiC-SR overcomes these limitations by redesigning the encoder as a modified U-net architecture (Ronneberger et al. 2015), and introducing a multiscale attention mechanism. This novel model allows RefHiC-SR to handle large patches in Hi-C matrices while still benefiting from a reference panel.

## 2 Materials and methods

### 2.1 RefHiC-SR model architecture

**Figure 1.**
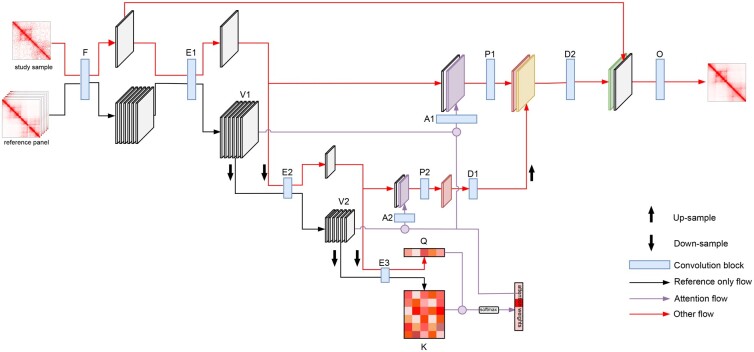
RefHiC-SR architecture. Overview of the RefHiC-SR neural network for enhancing Hi-C contact maps.

The RefHiC-SR network follows the U-net architecture (Ronneberger et al. 2015; Fig. 1), originally introduced for image segmentation, to enhance the expressiveness of latent features produced by encoding blocks and enable effective handling of large patches (i.e. 200 × 200). It contains (i) a low-level feature extraction block (F) that transforms a Hi-C matrix to multichannel features, (ii) an output block (O) that transforms multi-channel features to an enhanced Hi-C matrix, (iii) multiscale encoding blocks (E1, E2, and E3) that transform low-level features to high-level features at different scales (i.e. keys, values, and query in attention Equations 1 and 2), (iv) multiscale decoding blocks (D1 and D2) that transform features at different scales, and (v) attention convolutional blocks (A1 and A2) and projecting layers (P1 and P2) that increasing the model complexity and reduces hidden feature dimensions. To inject information from reference samples into the U-net computation graph, in the forward pass, blocks F and E1–E3 compute multiscale embeddings for the study sample and for the *n* reference samples. We denote parts of these embeddings as V1 and V2 (values), K (keys), and Q (query). We then compute combined multiscale representations of all reference samples from these embeddings with an attention mechanism. Finally, we replace skip connections in U-net (Ronneberger et al. 2015) with a concatenation of the study sample’s embedding and a transformed attention output at the same scale.

F takes an input of dimension *w* × *w*, where *w* is the window size (w=200 at 5 kb resolution) and projects the input to a w×w×d embedding (d=24). It is built with one rectified linear unit activated (ReLU-activated) convolution layer with *d* 9 × 9 filters. E1, E2, and E3 are three consecutive encoding blocks linked by a max pooling operator with a 2 × 2 kernel and a stride of 2 (i.e. downsampling by 50%). E1, E2, and E3 extract multi-scale features from the input contact maps. E1 is built with two ReLU-activated convolution layers with *d* 3 × 3 filters and a dropout layer with rate = 0.2 between convolution layers. It takes an input of dimension w×w×d and produces an output of the same dimension. E2 is built with the same layers as E1, but it takes an input of dimension *w*/2 × *w*/2 × *d* and produces an output of the same dimension. E3 starts with a batch normalization layer and ends with a flatten layer. It contains three convolution layers: The first two contain *d* 3 × 3 filters, and the last contains one 3 × 3 filter. The first convolution layer in E3 is followed by a dropout layer with rate 0.2 and a max pooling operator with a 2 × 2 kernel and a stride of 2. We did not use batch normalization in blocks F, E1, and E2 as we observed it introduces artifacts in enhanced contact maps. E3 takes as input the downsampled output of E2 and produces embedding of dimension 1 × (w/8)^2^. The attention module (i.e. purple module) takes as query (Q) and keys (K) the outputs of E3, uses as values (V1 and V2) the outputs of E1 and E2 for the *n* reference samples. We define the attention weights α=softmax$(QK^{T})\in R^{1\times n}$, where $\alpha_{j}$ represents the relative amount of attention paid to sample *j* in our reference panel when analyzing the study sample. The attention output $a_{1}$ and $a_{2}$ at two levels are computed as: where A1 and A2 are convolution blocks for attention outputs configured similarly to E1 and E2 but preceded by layer normalization. P1 and P2 are built with one ReLU-activated convolution layer with one 3 × 3 filter. They project the concatenation of study-sample embeddings (produced by E1 and E2) and attention embeddings (i.e. $a_{1}$ and $a_{2}$) to embeddings with *d* channels. D1 and D2 are built similarly to E2 and E1. D1 takes as input the output of P2; meanwhile, D2 takes as input the concatenation of the output of P1 and the upsampled output of D1. O is built with two ReLU-activated convolution layers with 3 × 3 filters. It projects the concatenation of the output of F for the study sample and the output of D2 to an enhanced contact map.


(1) $$\begin{array}{c} a_{1}=\text{softmax}(QK^{T})V_{1}+A1(\text{softmax}(QK^{T})V_{1}) \end{array}$$

(2) $$a_{2}=\text{softmax}(QK^{T})V_{2}+A2(\text{softmax}(QK^{T})V_{2})$$

### 2.2 Hi-C data and preprocessing

RefHiC-SR’s input for an individual sample (i.e. study or reference samples) is defined as a matrix in the shape of *w* × *w*, corresponding to the region of interest with a window of size *w*. *w* is a hyperparameter set to w=200 at 5 kb resolution. We trained RefHiC-SR with ICE-normalized iterative correction and eigenvector decomposition normalized (ICE-normalized) Hi-C contact maps. RefHiC-SR can also take raw data as input, but using raw data directly can lead to worse prediction due to systematic bias. For model training, we used Hi-C data downsampled from the combined GM12878 Hi-C contact map (Rao et al. 2014). The length of TADs and the distance between chromatin loop anchors are usually within 3 Mb. Thus, we restricted our analysis to contact pairs separated by at most 3 Mb. For inference of an entire chromosome, we will first split a contact map into partially overlapping squares $X_{i,j}$ with width *w* indicated by top-left corner (i,j) and step w−20, where j≥i. We then apply the trained model to enhance each square. The width of the predicted squares is also *w*. Finally, we extract a (w−20)×(w−20) matrix by trimming each side to address discontinuity between adjacency matrices. The full-chromosome super-resolution contact map is obtained by tiling the super-resolution submatrices.

### 2.3 Model training

We trained, evaluated, and tested RefHiC-SR on contact maps downsampled from the combined GM12878 Hi-C data. We used chr11 and chr12 for validation, Chromosomes 15–17 for testing, and the rest of autosomes for training. After preparing the input data as mentioned above, we collected 6918, 798, and 813 200 × 200 blocks for training, validation, and testing. RefHiC-SR takes submatrices from the study and reference samples as input in the forward pass. To reduce training computation, we sampled 10 reference samples for each example in each epoch independently. During evaluation, we used all samples in the reference panel. We trained models with a batch size of 46 for 2000 epochs on an RTX6000 GPU and used AdamW optimizer (Loshchilov and Hutter 2017; weight_decay = 0.1; learning rate = 1*e*−3). We also used early stopping to prevent overfitting. In the first five training epochs, we warmed up the learning rate from 0 to the initial learning rate (i.e. 1*e*−3) and then reduced the learning rate to 1*e*−6 in the first 95% epochs using the cosine annealing learning rate scheduler. Following RefHiC (Zhang and Blanchette 2022), we performed data augmentation by downsampling Hi-C contact maps during training. This transformation preserves topological structures in Hi-C data. Briefly, we downsampled Hi-C training data and stored them on disk in advance. During training, we randomly selected one contact map from these downsampled contact maps for each training example in each epoch independently. We used L1 loss to train RefHiC-SR. It is simple and less prone to be over-smooth.

### 2.4 Contrastive pretraining

We pretrained low-level feature extraction block (F) and encoding blocks (E1–E3) by supervised contrastive learning (Gao et al. 2021) using Hi-C contact maps downsampled from the combined GM12878 Hi-C data. For each training example, we defined items extracted from the downsampled contact maps at the same region as similar items and all Hi-C contact map submatrices in the same batch at other regions as negative items. We aimed to train these layers such that the distances of embeddings produced by E3 for a training example and its similar items are as close as possible while of embeddings between a training example and its negative items are as far as possible. Following (Gao et al. 2021), we defined the loss for training instance *i* as cross-entropy with in-batch negatives: where $h_{i}$, $h_{i}^{+}$, and $h_{j}^{-}$ are embeddings: $h_{i}$ represents item *i*, $h_{i}^{+}$ represents one of item *i’*s similar items, $h_{j}^{-}$ represents an item with a label different from *i* (i.e. negative item). τ is a temperature that controls training, and we set it as 1. We pretrained the encoder for 20 epochs with the LARS using Adam as a base optimizer. We set batch size to 46 and learning rate to 1*e*−3 during training.


(3) li=−log esim(hi,hi+)/τesim(hi,hi+)/τ+∑j=iesim(hi,hj−)/τ

### 2.5 Evaluation metrics

We extensively compared the performance of RefHiC-SR with alternative tools using different metrics, including MSE, mean absolute error (MAE, a.k.a. L1), Pearson correlation coefficient (PCC), Spearman rank correlation coefficient (SRCC), and widely used metric in super-resolution image analysis, including structural similarity index measure (SSIM) score, and peak signal to noise ratio (PSNR) score (Hong et al. 2019) for each of the 200 × 200 submatrix predicted by each tool.

We compared super-resolution to high-resolution Hi-C contact maps with HiCRep (Lin et al. 2021). HiCRep measures the reproducibility of two Hi-C experiments by computing a stratified correlation coefficient (PCC) for two contact maps. Its score ranges from −1 to 1 where a high value indicates high reproducibility. In addition to using PCC to compute HiCRep scores, we also computed HiCRep scores with SRCC.

### 2.6 Hi-C data downsampling and Hi-C reference panel

We used the original and six-level downsampled data of the combined Hi-C contact map for GM12878 cells obtained from Rao et al. (2014) to train RefHiC-SR. The reference panel that contains 30 human Hi-C contact map are used to train and evaluate RefHiC-SR. We excluded samples that belong to the study sample’s cell type from the reference panel to prevent potential data leakage.

### 2.7 Hyperparameter tuning

We first evaluate the performance of different convolution blocks in RefHiC-SR by adjusting convolution layer numbers in each block and adding residual connections. We configured most blocks with two convolution layers. Following (Zhang et al. 2018; Hong et al. 2019), we used 3 × 3 filters in all internal convolution layers, but tested different filter sizes (i.e. 3 × 3, 5 × 5, 9 × 9, and 13 × 13) for the first and last convolution layers. We compared validation errors and determined the optimal filter size as 9 × 9 for both layers. We also compared RefHiC-SR trained with MSE and L1 loss. We observed the model trained with MSE loss overly smooth predictions.

### 2.8 Contact map enhancement with alternative tools

We re-trained HiCPlus, HiCCNN, and DeepHiC with the same data as we used to train RefHiC-SR. Following previous work (Zhang et al. 2018; Hong et al. 2019; Liu and Wang 2019), we trained each model by splitting Hi-C contact maps into 40 × 40 blocks. To train HiCPlus and HiCNN, we adjusted learning rate and used early stopping to prevent overfitting and set other hyperparameters as default. We trained HiCPlus (Zhang et al. 2018) for 30 000 epochs with a learning rate of 1*e*−3 and a batch size of 256. We trained HiCNN (Liu and Wang 2019) and DeepHiC (Hong et al. 2019) for 1000 epochs with a learning rate of 1*e*−4 and a batch size of 256. The maximum training epochs we used are much larger than their original setting, and training losses indicated all models were converged. DeepHiC’s discriminator is too strong to provide gradients to the generator with its original training procedure in our experiment. We changed the discriminative loss weight to 0.0001 and updated the discriminator every ten epochs. Once trained, we applied each model to 200 × 200 overlapped blocks to enhance a whole contact map. Same as RefHiC-SR, we cropped the prediction into a matrix of 180 × 180.

## 3 Results

We introduce RefHiC-SR, a reference panel-informed DL approach for enhancing Hi-C contact maps. Similar to RefHiC (Zhang and Blanchette 2022), it utilizes a reference panel containing 30 high-quality Hi-C datasets from multiple human cell types (Supplementary Table S1) and employs an attention mechanism to determine which reference samples are most relevant for a given *w* × *w* region of the contact map of the study sample. The enhanced contact map at a given region is then inferred based on a combination of the study sample and the attention-weighted reference samples. RefHiC-SR takes as input a typical ICE normalized (Imakaev et al. 2012; Abdennur and Mirny 2020) moderate-coverage (sparse) Hi-C contact map and outputs a high-coverage (dense) contact map prediction. Both input and output contact maps are at high resolution (i.e. 5-kb bins). The resulting prediction is referred to as enhanced or super-resolution contact maps. We divided the human autosomes into a training set (Chromosomes 1–10, 13, 14, and 18–22), a validation set (Chromosomes 11 and 12), and a test set (Chromosomes 15–17). All results reported here pertain only to the three test chromosomes. Although we trained RefHiC-SR on GM12878 cells, the model learned is not cell-type specific. We will demonstrate in a later section that we can use the same model to enhance Hi-C data of other cells.

RefHiC-SR’s neural network takes as input a matrix of 200 bins by 200 bins, and outputs a super-resolution matrix of the same dimension. When applying a trained model to full-chromosome contact map enhancement, we extract from the output matrix a 180 × 180 matrix by trimming each side to address discontinuity between adjacency matrices. The full-chromosome super-resolution contact map is obtained by tiling the super-resolution submatrices. Other super-resolution tools were applied in the same manner.

### 3.1 RefHiC-SR accurately enhances low-coverage contact maps

We first assessed the contact map enhancement performance of RefHiC-SR, in comparison to three approaches: HiCPlus, HiCNN, and DeepHiC on test Chromosomes 15–17 of GM12878 cells. Each model represents one of the three types of DL models in contact map enhancement (i.e. shallow model, deep model, and GAN), with DeepHiC featured as the state-of-the-art model in several studies. We used as input a 5-kb resolution Hi-C dataset produced from 250-M valid read pairs, obtained by downsampling a Hi-C dataset for human GM12878 cells (Rao et al. 2014). This is equivalent to a 1/16 downsampling that most existing tools were trained and evaluated at. As existing models are trained from data at 10-kb resolution and with different normalization approaches, it is impractical to benchmark trained models on the same data at 5 kb. Thus, we retrained HiCPlus, HiCNN, and DeepHiC with the same set of training data as we used to train RefHiC-SR (see Section 2). The accuracy of the enhanced contact maps is assessed by comparing it to the full coverage contact map, using seven metrics: (i) MSE, (ii) MAE, (iii) PSNR (Liu et al. 2019), (iv) the SSIM (Hong et al. 2019), (v) the diagonal-wise PCC, (vi) the diagonal-wise SRCC, and (vii) the HiCRep score (Yang et al. 2017) for Hi-C data comparison.

**Figure 2.**
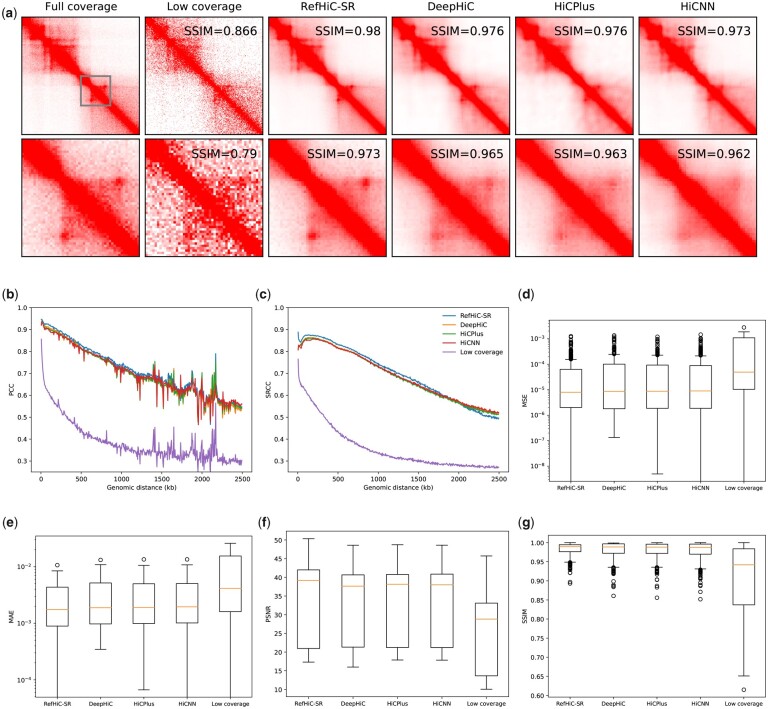
Comparison of RefHiC-SR and other tools on GM12878 Hi-C data (250-M valid read pairs, test Chromosomes 15–17). a. Examples of full coverage, low coverage, and enhanced contact maps on a 1-Mb genomic region (chr17:5000000–6000000) and a zoom in portion. Diagonal-wise PCC (b) and SRCC (c). Boxplots of MSE (d), MAE (e), PSNR (f), and SSIM (g) between full coverage and enhanced contact maps.

Figure 2a and Supplementary Fig. S1 illustrate the full coverage (target), low coverage (input), and enhanced contact maps and their differences on a typical 1-Mb genomic region (chr17:5000000–6000000). We observed that all enhanced contact maps better match the full coverage contact map than the low-coverage contact map does, with a slightly advantage for RefHiC-SR. RefHiC-SR and DeepHiC are better capturing fine-scale structures such as loops. We then compared the prediction quality on test Chromosomes 15–17. The diagonal-wise PCC and SRCC between the enhanced and full coverage contact maps (Fig. 2b and c andSupplementary Fig. S10), show that RefHiC-SR is comparable to or outperforms existing tools across all distance ranges. We then compared RefHiC-SR with existing tools at individual 180 × 180 submatrices. The distributions of MSE (Fig. 2d), MAE (Fig. 2e), PSNR (Fig. 2f), and SSIM (Fig. 2g) show that all tools achieve similar performance, with a slight advantage for RefHiC-SR. Finally, we compared the similarity of super-resolution and full coverage contact maps at the whole-chromosome level with HiCRep (Yang et al. 2017). Table 1 shows HiCRep scores for test chromosomes. It indicates that RefHiC-SR is among the best across test chromosomes.

**Table 1.** HiCRep scores between high-coverage and 1/16 downsampled (low coverage)/enhanced contact maps of GM12878 cells.^a^

	PCC			SRCC		
	chr15	chr16	chr17	chr15	chr16	chr17
RefHiC-SR	**0.898** ± **0.001**	**0.865** ± **0.001**	**0.877** ± **0.003**	**0.845** ± **3e**−**4**	**0.845** ± **3e−4**	0.824±0.001
DeepHiC	0.884 ± 6e−4	0.842 ± 0.001	0.863 ± 0.004	0.833 ± 5e−4	0.830±4e−4	0.820±9e−4
HiCNN	0.888 ± 6e−4	0.847 ± 0.001	0.867 ± 0.003	0.844 ± 4e−4	0.844±3e−4	**0.830** ± **8e−4**
HiCPlus	0.865 ± 4e−4	0.823 ± 0.001	0.845 ± 0.003	0.807 ± 5e−4	0.804±5e−4	0.792±0.001
Low coverage (input)	0.643 ± 0.001	0.632 ± 0.002	0.661 ± 0.002	0.559 ± 5e−4	0.564±4e−4	0.558±6e−4

a HiCRep scores are computed with PCC and SRCC metrics. We computed the standard deviations by repeating the analysis five times on data downsampled with different random seeds.

### 3.2 RefHiC-SR is robust to sequencing depths

**Figure 3.**
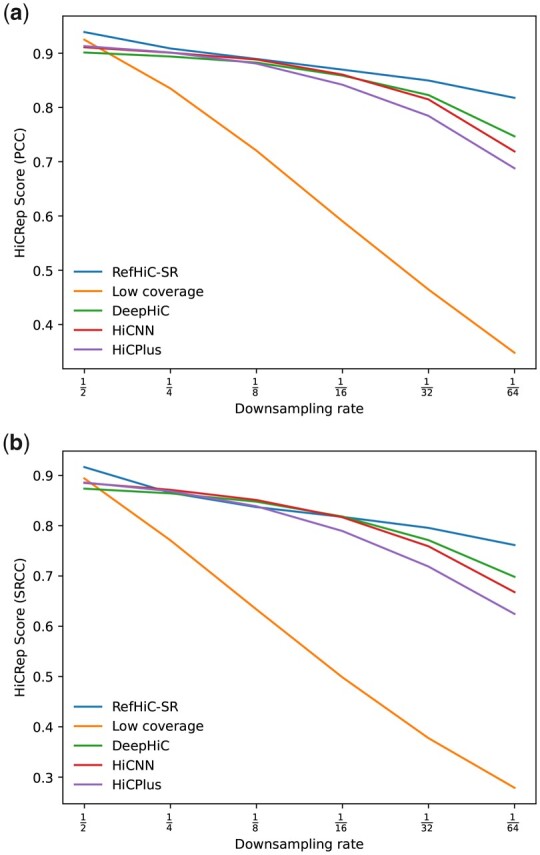
Average HiCRep scores from test Chromosomes 15–17 from the GM12878 cell line across downsampling ratios 1/2, 1/4…1/64. HiCRep scores are computed with PCC (a) and SRCC (b) metrics.

To benchmark RefHiC-SR’s ability to enhance contact maps from Hi-C data at different sequencing depths, we produced downsampled versions (i.e. 1/2, 1/4,…, 1/64, where 1/64 = 62.5 M valid read pairs) of the same GM12878 contact map (Rao et al. 2014) and applied RefHiC-SR and other tools to enhance contact map resolutions for test chromosomes. We evaluated the accuracy of enhanced contact maps by comparing them against the full coverage contact maps using HiCRep. Although lower sequencing depths led to less accurate enhancement for all tools (Fig. 3), RefHiC-SR was most robust to low sequencing depths, clearly outperforming other tools at very low coverage (1/32 = 125 M and 1/64 = 62.5 M). For Hi-C data containing <250 M valid read pairs, RefHiC-SR can produce predictions comparable to the second best tool using only half of the read pairs. To study the performance of each tool at the most extreme case (i.e. 1/64 downsampled data), we repeated the battery of tests originally performed at 1/16 = 250 M downsampled data (Supplementary Figs S2 and S10). We observed RefHiC-SR performed the best on all metrics.

### 3.3 RefHiC-SR performs well across cell types

Next, we aimed to assess the performance of RefHiC-SR and other tools, which were all trained on Hi-C data obtained from GM12878 cells, on data from other cell types. We applied each model to the enhancement of Hi-C data from IMR90 and K562 (Rao et al. 2014) cell lines (test Chromosomes 15–17 only). We used HiCRep, MAE, MSE, PSNR, and SSIM to evaluate model performance. Table 2 shows that RefHiC-SR outperformed other tools by achieving the highest HiCRep scores in both cells. Supplementary Figs S8 and S9 show that RefHiC-SR outperformed or was comparable to other tools in both cells as evaluated by super-resolution image analysis metrics.

**Table 2.** HiCRep scores between high- and low-coverage/enhanced contact maps of IMR-90 and K562 cells.^a^

	IMR90			K562		
	chr15	chr16	chr17	chr15	chr16	chr17
RefHiC-SR	**0.868**	**0.850**	**0.878**	**0.813**	**0.817**	**0.825**
DeepHiC	0.858	0.839	0.866	0.799	0.810	0.812
HiCNN	0.861	0.842	0.870	0.802	0.804	0.811
HiCPlus	0.858	0.840	0.866	0.812	0.817	0.824
Low coverage (input)	0.716	0.697	0.722	0.788	0.787	0.806

a HiCRep scores are computed with PCC metrics.

### 3.4 RefHiC-SR enables improved loop and TAD boundary annotation

RefHiC-SR and other resolution enhancement tools are meant to ease downstream analyses, such as TAD and loop annotation, by imputing missing signals in contact maps. Here we show that RefHiC-SR facilitates annotating TAD and loop with off-the-shelf annotation tools, without introducing many false positives.

**Figure 4.**
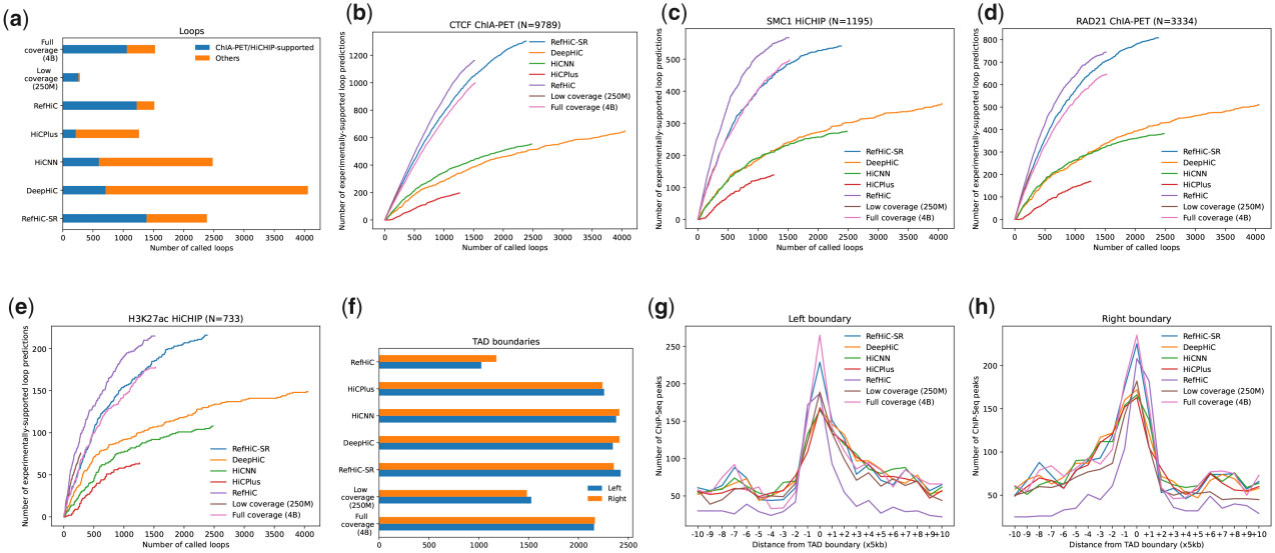
Comparison of loops and TADs annotated from low coverage, full coverage, and enhanced contact maps. (a) Number of loop annotations. (b–e) Number of ChIA-PET/HiCHIP-supported loop predictions compared against CTCF ChIA-PET (b), SMC1 HiCHIP (c), RAD21 ChIA-PET (d), and H3K27ac HiCHIP (e). Occupancy of ChIP-seq identified CTCF binding site as a function of distance to left (g) and right (h) boundary annotations.

We first assessed the ability of RefHiC-SR to produce enhanced maps that enable high-accuracy loop prediction. We applied Mustache (Roayaei Ardakany et al. 2020) to annotate loops from the original full coverage GM12878 contact map, the 1/16 downsampled contact map, and enhanced contact maps produced by different tools. For each analysis, we set the same Mustache 5% false discovery rate (FDR) cutoff, keeping other parameters as default, and sorted loops by Mustache-reported FDR. We also included predictions made by RefHiC (Zhang and Blanchette 2022) for comparison. The number of predicted loops is quite different among different inputs, with DeepHiC leading to the largest number of predictions (Fig. 4a). We then evaluated predicted loops by comparing them to loops identified by loop-targeting experimental data [ChIA-PET on CTCF (CCCTC-binding factor) (Tang et al. 2015) and RAD21 (ENCODE Project Consortium et al. 2012), and HiChiP on SMC1 (Mumbach et al. 2016) and H3K27ac (Mumbach et al. 2017)], allowing up to a 5-kb shift (Fig. 4b–e). When applied to enhanced contact maps, Mustache produced 1245 CTCF-supported loops, 761 RAD21-supported loops, 512 SMC1-supported loops, and 197 H3K27ac-supported loops from RefHiC-SR enhanced contact maps. These numbers exceed those obtained on enhanced maps produced by other tools by 25%–550%, and even those obtained on the full coverage data itself. The accuracy of loop annotated from RefHiC-SR enhanced contact maps is comparable to annotating loops from the full coverage data. In contrast, contact maps enhanced by alternative tools introduced a large number of false positive loop predictions. The combination of RefHiC-SR and Mustache was only beat by RefHiC slightly. We next evaluated the extent to which RefHiC-SR facilitates loop annotation from Hi-C contact maps containing different numbers of valid read pairs. Unexpectedly, coverage reduction leads to an increase in the number of loops being predicted on most tools’ enhanced maps (including RefHiC-SR enhanced, Supplementary Fig. S3). Supplementary Fig. S4 shows that as the coverage drops from 256 to 62.5 M valid read pairs, the number of experimentally supported predicted loops remains similar when Mustache is applied to RefHiC-SR enhanced contact maps, but drops significantly using enhanced maps produced by other super-resolution tools.

To evaluate RefHiC-SR’s usefulness for facilitating TAD annotation, we used RobusTAD (Dali et al. 2018) to annotate TAD boundaries from the same set of contact maps as above. We also included predictions made by RefHiC (Zhang and Blanchette 2022) for comparison. RefHiC made the least predictions. The number of predicted TAD boundaries is similar among full coverage Hi-C data and super-resolution inputs (Fig. 4f). Figure 4g and h shows that RobusTAD identified a similar total number of CTCF-supported TAD boundaries from RefHiC-enhanced and full coverage contact maps. In contrast, contact maps enhanced by alternative tools lead to fewer CTCF-supported TAD boundaries. Annotating TAD boundaries from contact maps enhanced from low-coverage data show that boundary annotation is robust to sequencing coverage (Supplementary Figs S5 and S6). Supplementary Fig. S6 shows that at very low coverage, RefHiC-SR can still help to identify a large number of CTCF-supported TAD boundaries.

We then repeated the analysis on Hi-C datasets for K562 and IMR-90 cells. Supplementary Figs S12 and S13 show RefHiC-SR outperformed alternative methods in both loop and TAD annotations.

### 3.5 RefHiC-SR implementation

RefHiC-SR is a Python program available at https://github.com/BlanchetteLab/RefHiC with scripts to reproduce our experiments. We implemented the neural network with the PyTorch library (Paszke et al. 2019). RefHiC-SR can run on either a CPU or GPU, but it performs three times faster on GPU. RefHiC-SR requires at least 3 GB of storage space for saving reference panel data and at least 15-GB RAM for loading reference samples during prediction. RefHiC-SR is efficient and can process the longest human chromosome within 3 min when run on a GPU.

## 4 Discussion

Here, we present RefHiC-SR, a DL framework that utilizes a reference panel to facilitate enhancing Hi-C data resolution for a given study sample. In contrast, existing contact map enhancement algorithms are exclusively study-sample based, and hence their ability to reliably enhance contact maps from typical sequencing depth Hi-C data is limited. Our extensive evaluation demonstrated that RefHiC-SR outperforms existing tools in datasets ranging from very high to very low sequencing coverage, with the most striking improvements observed in the latter case. RefHiC-SR also outperformed a simple global similarity-based baseline (Supplementary Note S2), indicating the necessity of designing this model to incorporate the reference panel. Although RefHiC-SR is a machine-learning model trained primarily on GM12878 Hi-C data, the same trained model is effective on different cell types, and at different levels of coverage. Comparison between RefHiC-SR and a Baseline model similar to RefHiC-SR but lacking a reference panel shows RefHiC-SR’s superior to the introduction of a reference panel (Supplementary Note S1). The super-resolution contact maps predicted by RefHiC-SR are ready for downstream analysis and do not introduce a significant number of false positives. In contrast, other enhancement tools often introduce false positive annotations in downstream analysis.

Across the different subfields of data-driven biology, researchers have developed many reference panel enabled approaches to aid the analysis of a study sample. RefHiC-SR is the first approach to enable this type of reference panel based analysis of 3D contact map enhancement. In addition, RefHiC-SR is the only contact map enhancement model that is robust to low sequencing coverage. We believe RefHiC-SR has the potential to become an essential method for enhancing Hi-C contact maps, paving the way to further our understanding of 3D genome organization and functional implications at a finer scale.

## Supplementary Material

btad266_Supplementary_Data

## Data Availability

The data underlying this article are available in zenodo, at https://dx.doi.org/10.5281/zenodo.7761968.
