# Supplementary material for: Reference panel-guided super-resolution inference of Hi-C data
Source: Bioinformatics. 2023 Jun 30;39(Suppl 1):i386–93. doi: 10.1093/bioinformatics/btad266 (PMC10311349; doi:10.1093/bioinformatics/btad266)
Supplement: btad266_Supplementary_Data [file btad266_supplementary_data.pdf]

Supplementary Information
Reference panel guided super-resolution inference of Hi-C data

Yanlin Zhang[1] and Mathieu Blanchette[1,*]

[1]*School of Computer Science, McGill University, Montréal, Québec, H3A 0E9, Canada*
[*]*Correspondence: blanchem@cs.mcgill.ca*

Supplementary Note S1. Comparison with a U-Net Baseline

We built a U-Net model named Baseline to demonstrate the superior performance achieved by RefHiC-SR is attributed to the use of a reference panel. This Baseline model is similar to RefHiC-SR but does not use a reference panel. We followed RefHiC-SR's training procedure to train Baseline. Supplementary Fig. S4,S6,S7 show that RefHiC-SR outperformed Baseline in enhancing contact maps containing different numbers of valid read pairs. These results suggest that RefHiC-SR benefits from the introduction of a reference panel.

Supplementary Note S2. RefHiC-SR outperforms a simple top-K averaging baseline

Although RefHiC-SR has demonstrated outstanding performance when evaluated by various methods, it remains uncertain whether this superiority is attributable to the proposed local similarity-based U-Net model. It is interesting to investigate the performance of predicting as the average of the study sample and top-K most similar Hi-C reference samples. To establish a baseline for comparison, we employed a method, **Baseline (top 5)**, based on the top 5 most similar reference samples, which were identified by comparing the study sample against each reference sample using HiCRep. Subsequently, we calculated the average interaction frequency of the study sample and the selected reference samples to make our prediction. Supplementary Fig. S11 shows that Baseline (top 5) is much worse than RefHiC-SR.

ID	Sample	Valid read pairs	Source
HIC00001	22Rv1 (prostate cancer cell line)	685096962	[5]
HIC00002	293TRex-Flag-BRD4-NUT-HA	1660296754	[15]
HIC00007	BLaER (lymphoblastic leukemia cell line)	406322404	[17]
HIC00041	HCT-116 (colorectal cancer cell line)	603179292	[14]
HIC00067	HeLa Kyoto cell, MboI G1 sync control	1045885928	[19]
HIC00090	HepG2 (hepatocellular carcinoma cell line)	1759654311	[3]
HIC00091	HL60/S4 (neutrophil-like Myeloid leukemia cell line)	478434139	[8]
HIC00113	Nalm6 (B cell precursor leukemia cell line)	816274711	[18]
HIC00168	WI38_RAF (WI-38hTERT/GFP-RAF1-ER)	602556180	[16]
HIC00172	Embryonic stem cell, Cardiomyocyte differentiation : hESCs (day 0)	1914642484	[20]
HIC00183	teloHAEC (endothelial cell line)	911486437	[10]
HIC00200	Naïve human embryonic stem cells	731906045	[1]
HIC00203	GM23248 (primary skin fibroblasts)	1797370277	[12]
HIC00221	MDM (monocyte-derived macrophages)	590449106	[7]
HIC00269	Astrocytes of the cerebellum primary cell	430822244	[3]
HIC00273	HAP1 (near-haploid cell line)	413436528	[6]
HIC00280	Purified human germinal center B cells	426222299	[2]
HIC00287	Liver	447028100	[11]
HIC00295	Thymus	507309033	[11]
HIC00296	H1 Embroynic Stem Cell	989388439	[4]
HIC00310	A549 00h 100 nM dexamethasone	1548684355	[3]
HIC00318	HUVEC	438880295	[13]
HIC00319	IMR90	1053932182	[13]
HIC00320	K562	880877579	[13]
HIC00321	KBM7	877658969	[13]
HIC00322	NHEK	653628335	[13]
HIC00337	Gastric tissue	426476775	[9]
HIC00343	Left Ventricle	547477074	[9]
HIC00354	Spleen	490487515	[9]
HIC00360	GM12878	1994319522	[13]

Supplementary Table S1. Human reference panel

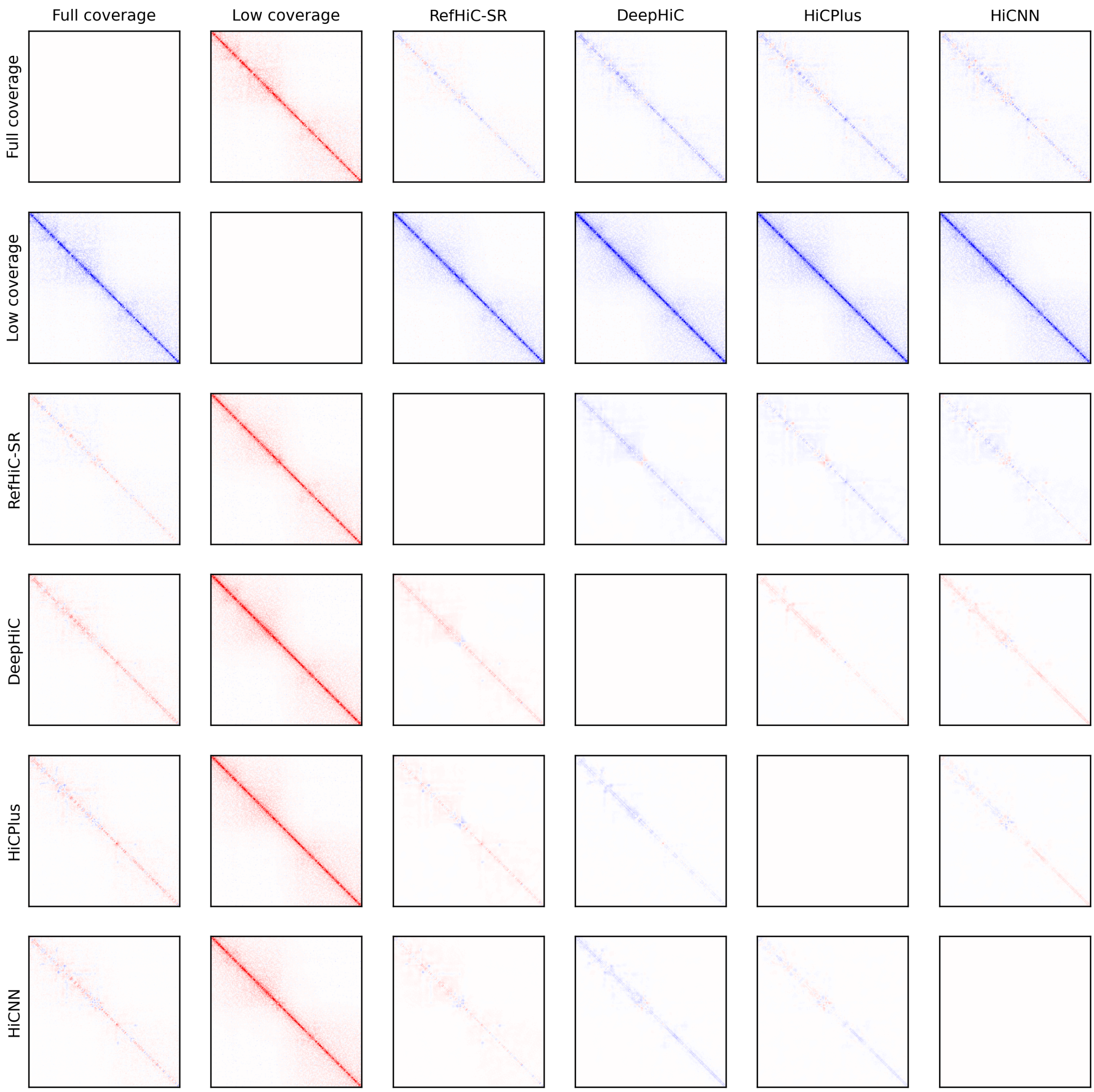

Supplementary Figure S1. Pairwise difference among low-coverage, full-coverage, and enhanced contact maps on a 1 Mb genomic region (chr17:5000000-6000000). We clipped values to the range of [-0.5,0.5] for better visualization.

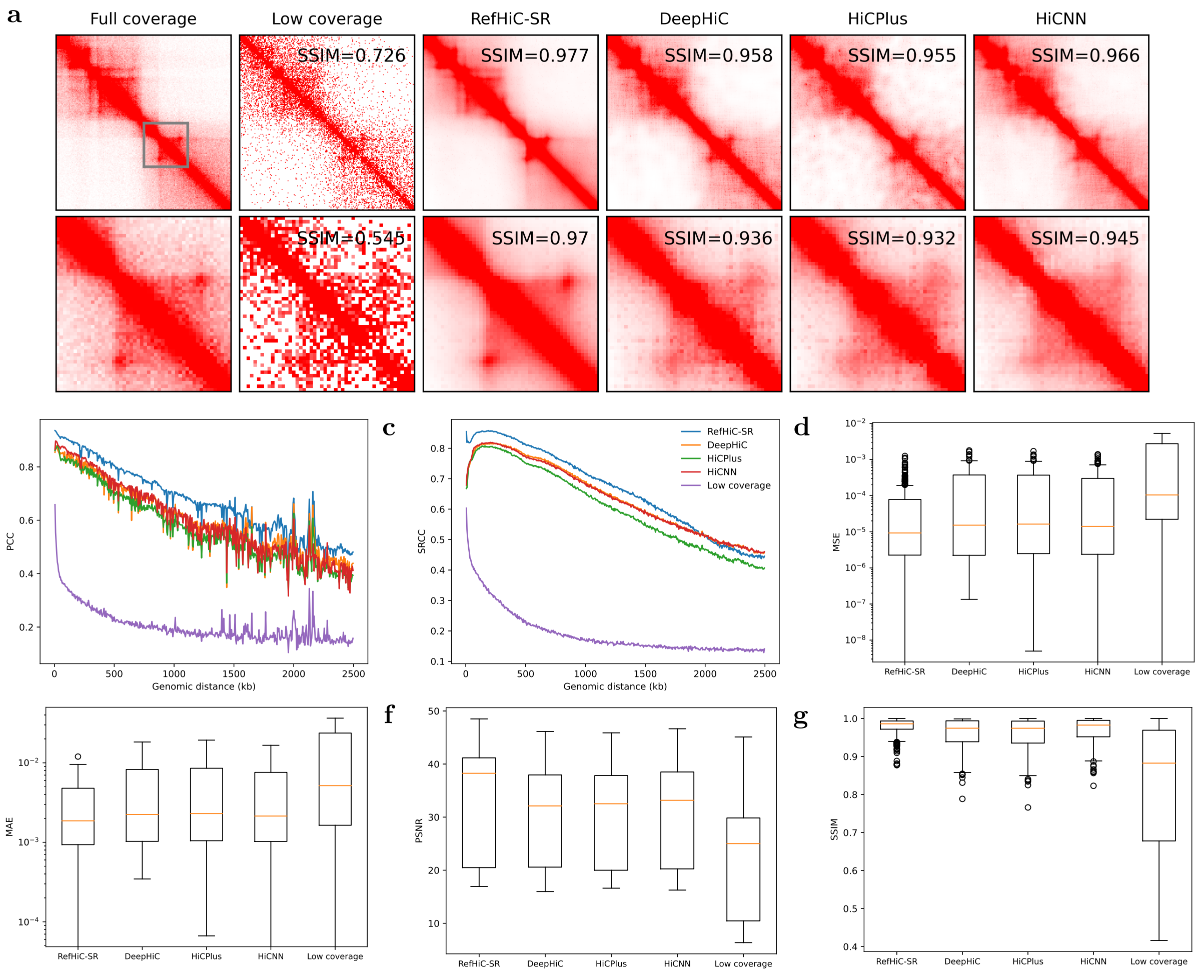

Supplementary Figure S2.Comparison of RefHiC-SR and other tools on GM12878 Hi-C data (62.5M valid read pairs, test chromosomes 15-17). a. Examples of low-coverage, full-coverage and enhanced contact maps on a 1 Mb genomic region (chr17:5000000-6000000). Diagonal-wise PCC (b) and SRCC (c). Boxplots of MSE (d), MAE (e), PSNR (f), and SSIM (g) between full-coverage and enhanced contact maps.

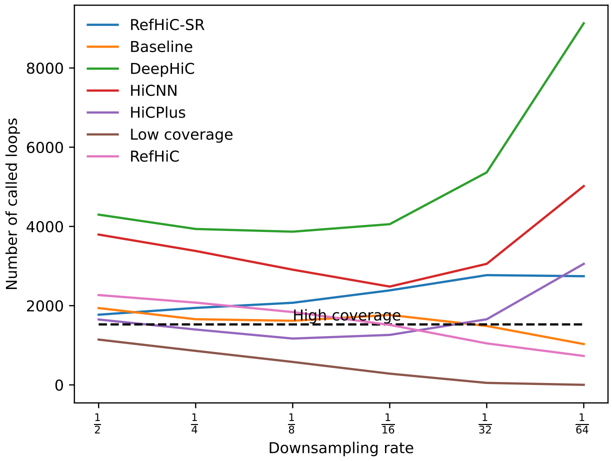

Supplementary Figure S3. Number of called loops predicted from data across different downsampling rates.

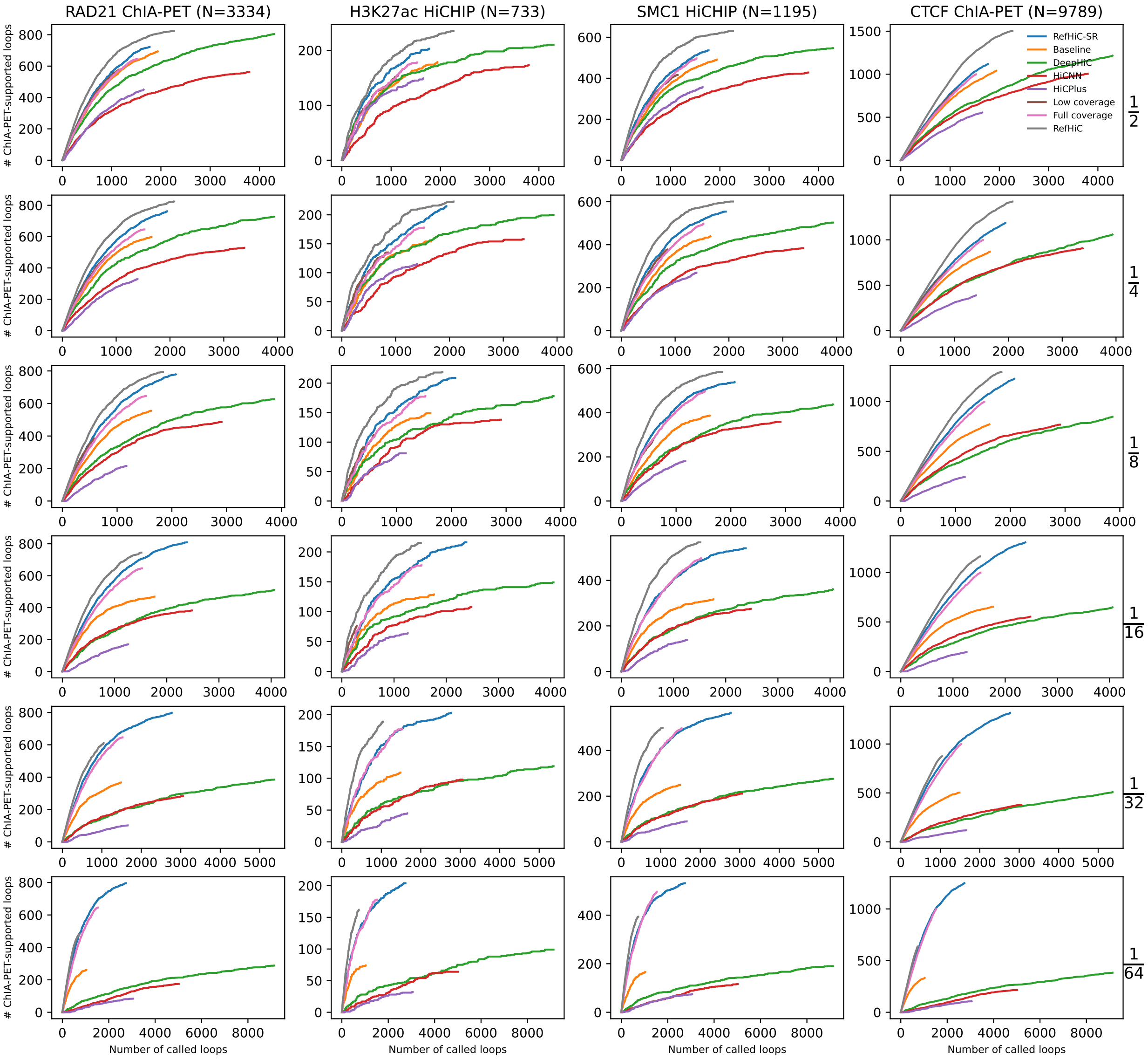

Supplementary Figure S4. Comparison of loops annotated from low-coverage, full-coverage, and enhanced contact maps. This figure is similar to Fig. 3b-e but for contact maps at different downsampling rates.

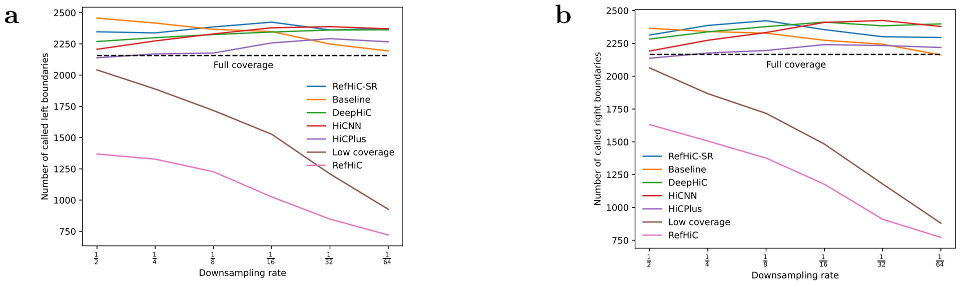

Supplementary Figure S5. Number of called left (a) and right (b) boundaries predicted from data across different downsampling rates.

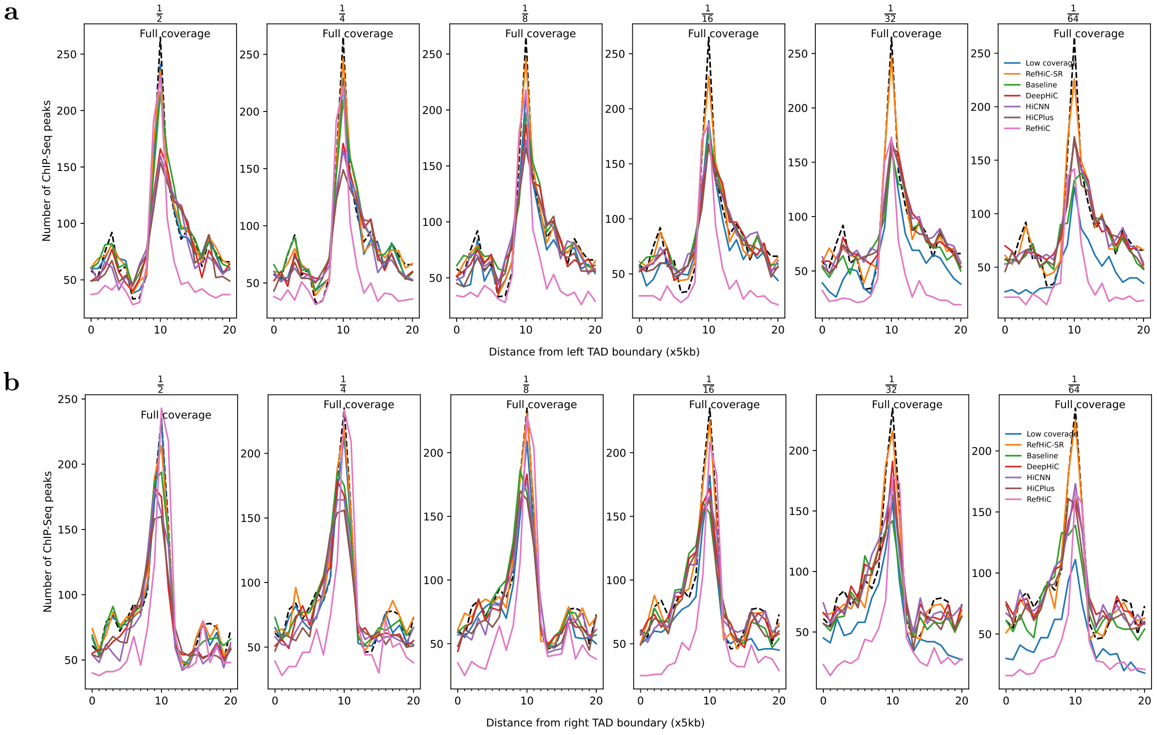

Supplementary Figure S6. Comparison of TADs annotated from low-coverage, full-coverage, and enhanced contact maps. This figure is similar to Fig. 3b-e but for contact maps at different downsampling rates.

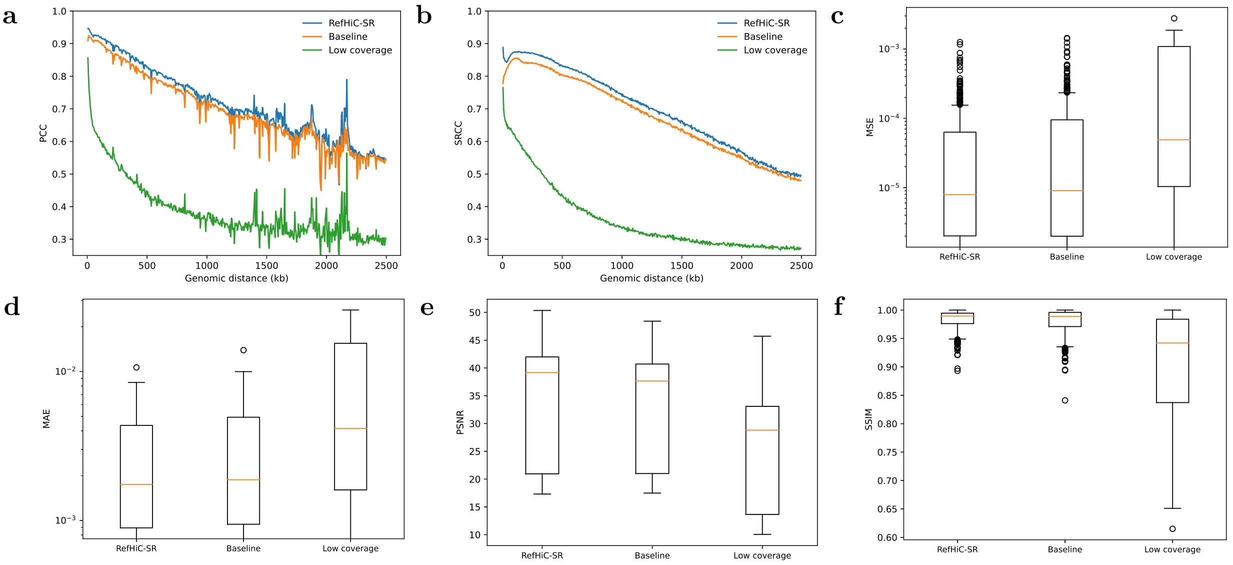

Supplementary Figure S7. Comparison of RefHiC-SR and Baseline on GM12878 Hi-C data (250M valid read pairs, test chromosomes 15-17). These figures are similar to Fig. 1 but compared RefHiC-SR against Baseline (Suppl. Note 1).

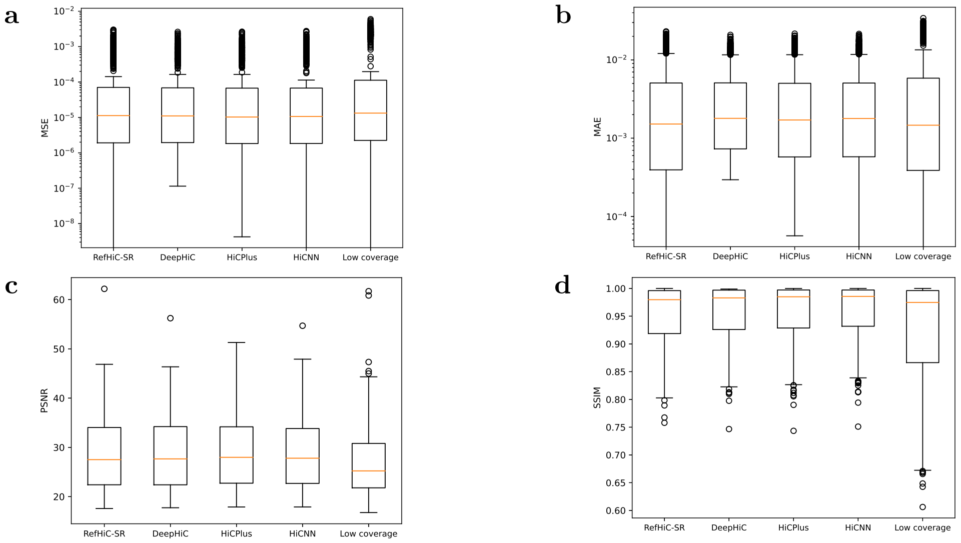

Supplementary Figure S8. Comparison of RefHiC-SR and other tools on IMR-90 Hi-C data (test chromosomes 15-17). Boxplots of MSE (a), MAE (b), PSNR (c), and SSIM (d) between full-coverage and enhanced contact maps.

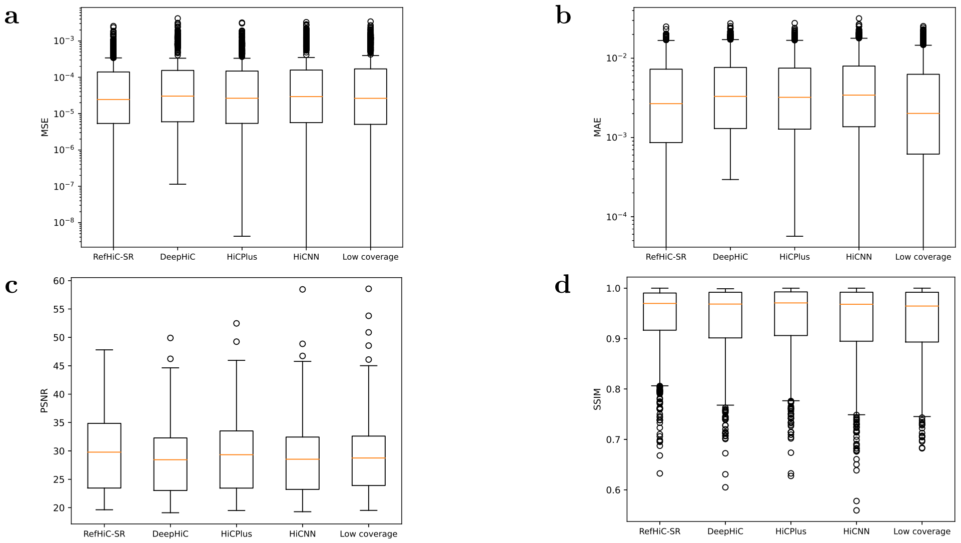

Supplementary Figure S9. Comparison of RefHiC-SR and other tools on K562 Hi-C data (test chromosomes 15-17). Boxplots of MSE (a), MAE (b), PSNR (c), and SSIM (d) between full-coverage and enhanced contact maps.

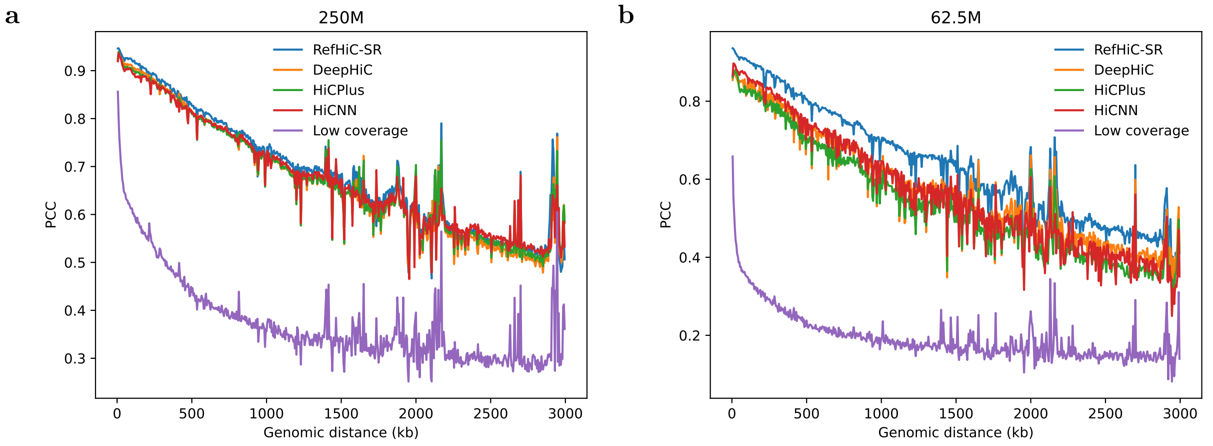

Supplementary Figure S10. Comparison of RefHiC-SR and other tools on GM12878 Hi-C data (test chromosomes 15-17, within 3Mb distance). Diagonal-wise PCC between the full coverage contact map and a contact map enhanced from a Hi-C dataset that containing 250M valid read pairs (a), and 62.5M valid read pairs (b). These are the same figures as Fig. 2b and Suppl. Fig. 2b, but including more long range interactions.

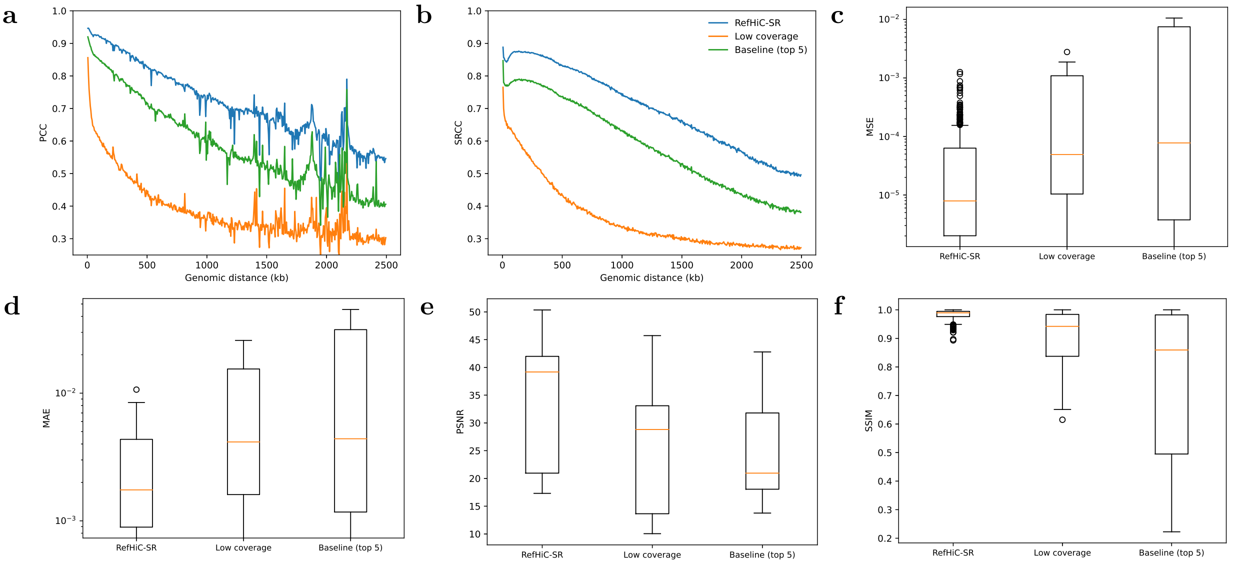

Supplementary Figure S11. Comparison of RefHiC and Baseline (top 5) described in Supplementary Note 2 on GM12878 HiC data (500M valid read pairs). Diagonal-wise PCC (a) and SRCC (b). Boxplots of MSE (c), MAE (d), PSNR (e), and SSIM (f) between full coverage and enhanced contact maps.

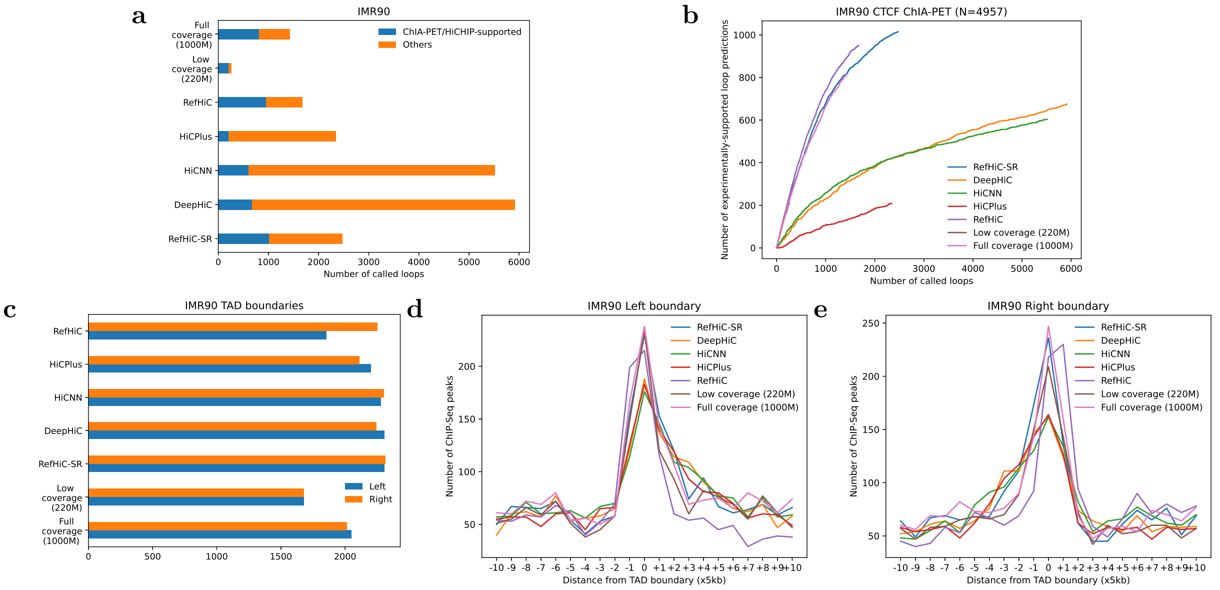

Supplementary Figure S12. Comparison of loops and TADs annotated from low coverage, full coverage, and enhanced contact maps for human IMR-90 cells. (a) Number of loop annotations. (b) Loop predictions compared against CTCF ChIA-PET. (c) Number of TAD boundary annotations. Occupancy of ChIP-seq identified CTCF binding site as a function of distance to left (d) and right (e) boundary annotations.

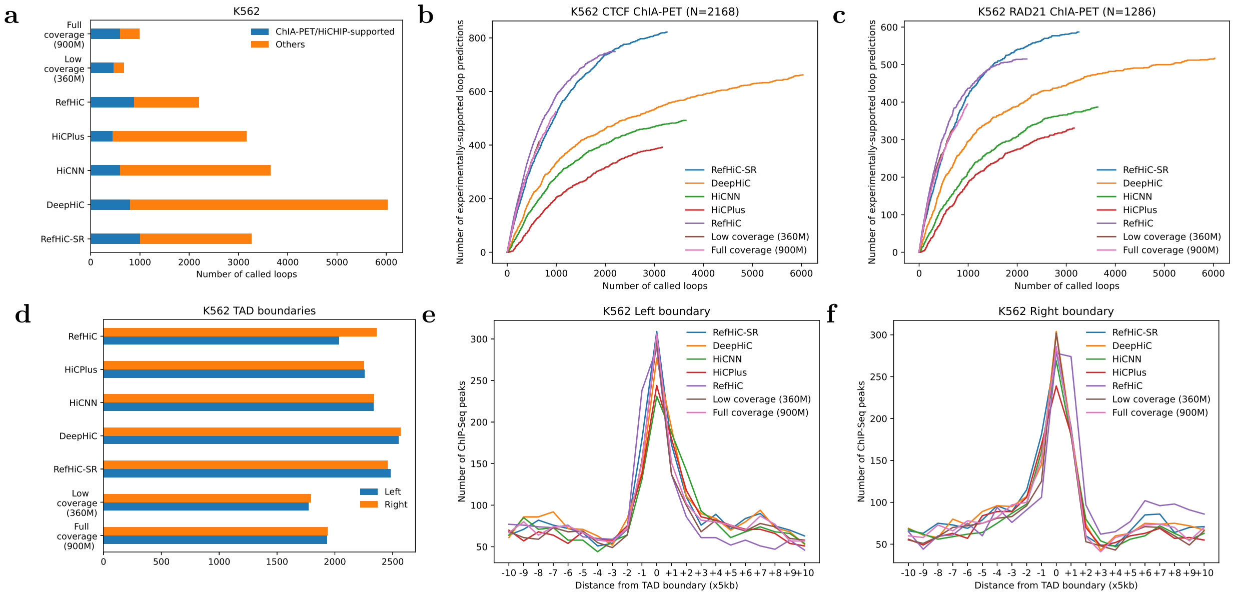

Supplementary Figure S13. Comparison of loops and TADs annotated from low coverage, full coverage, and enhanced contact maps for human K562 cells. (a) Number of loop annotations. Loop predictions compared against CTCF ChIA-PET (b) and RAD21 ChIA-PET (c). (d) Number of TAD boundary annotations. Occupancy of ChIP-seq identified CTCF binding site as a function of distance to left (e) and right (f) boundary annotations.

References

[1] S. L. Battle, N. Doni Jayavelu, R. N. Azad, J. Hesson, F. N. Ahmed, E. G. Overbey, J. A. Zoller, J. Mathieu, H. Ruohola-Baker, C. B. Ware, and R. D. Hawkins. Enhancer Chromatin and 3D Genome Architecture Changes from Naive to Primed Human Embryonic Stem Cell States. *Stem Cell Reports*, 12(5):1129–1144, 05 2019.

[2] K. L. Bunting, T. D. Soong, R. Singh, Y. Jiang, W. Béguelin, D. W. Poloway, B. L. Swed, K. Hatzi, W. Reisacher, M. Teater, O. Elemento, and A. M. Melnick. Multi-tiered Reorganization of the Genome during B Cell Affinity Maturation Anchored by a Germinal Center-Specific Locus Control Region. *Immunity*, 45(3):497–512, 09 2016.

[3] ENCODE Project Consortium et al. An integrated encyclopedia of dna elements in the human genome. *Nature*, 489(7414):57, 2012.

[4] J. R. Dixon, I. Jung, S. Selvaraj, Y. Shen, J. E. Antosiewicz-Bourget, A. Y. Lee, Z. Ye, A. Kim, N. Rajagopal, W. Xie, Y. Diao, J. Liang, H. Zhao, V. V. Lobanenkov, J. R. Ecker, J. A. Thomson, and B. Ren. Chromatin architecture reorganization during stem cell differentiation. *Nature*, 518(7539):331–336, Feb 2015.

[5] Y. Guo, A. A. Perez, D. J. Hazelett, G. A. Coetzee, S. K. Rhie, and P. J. Farnham. CRISPR-mediated deletion of prostate cancer risk-associated CTCF loop anchors identifies repressive chromatin loops. *Genome Biol*, 19(1):160, 10 2018.

[6] J. H. I. Haarhuis, R. H. van der Weide, V. A. Blomen, J. O. Yáñez-Cuna, M. Amendola, M. S. van Ruiten, P. H. L. Krijger, H. Teunissen, R. H. Medema, B. van Steensel, T. R. Brummelkamp, E. de Wit, and B. D. Rowland. The Cohesin Release Factor WAPL Restricts Chromatin Loop Extension. *Cell*, 169(4):693–707, 05 2017.

[7] S. Heinz, L. Texari, M. G. B. Hayes, M. Urbanowski, M. W. Chang, N. Givarkes, A. Rialdi, K. M. White, R. A. Albrecht, L. Pache, I. Marazzi, A. García-Sastre, M. L. Shaw, and C. Benner. Transcription Elongation Can Affect Genome 3D Structure. *Cell*, 174(6):1522–1536, 09 2018.

[8] E. C. Jacobson, J. K. Perry, D. S. Long, A. L. Olins, D. E. Olins, B. E. Wright, M. H. Vickers, and J. M. O'Sullivan. Migration through a small pore disrupts inactive chromatin organization in neutrophil-like cells. *BMC Biol*, 16(1):142, 11 2018.

[9] I. Jung, A. Schmitt, Y. Diao, A. J. Lee, T. Liu, D. Yang, C. Tan, J. Eom, M. Chan, S. Chee, Z. Chiang, C. Kim, E. Masliah, C. L. Barr, B. Li, S. Kuan, D. Kim, and B. Ren. A compendium of promoter-centered long-range chromatin interactions in the human genome. *Nat Genet*, 51(10):1442–1449, 10 2019.

[10] S. Lalonde, V. A. Codina-Fauteux, S. M. de Bellefon, F. Leblanc, M. Beaudoin, M. M. Simon, R. Dali, T. Kwan, K. S. Lo, T. Pastinen, and G. Lettre. Integrative analysis of vascular endothelial cell genomic features identifies AIDA as a coronary artery disease candidate gene. *Genome Biol*, 20(1):133, 07 2019.

[11] D. Leung, I. Jung, N. Rajagopal, A. Schmitt, S. Selvaraj, A. Y. Lee, C. A. Yen, S. Lin, Y. Lin, Y. Qiu, W. Xie, F. Yue, M. Hariharan, P. Ray, S. Kuan, L. Edsall, H. Yang, N. C. Chi, M. Q. Zhang, J. R. Ecker, and B. Ren. Integrative analysis of haplotype-resolved epigenomes across human tissues. *Nature*, 518(7539):350–354, Feb 2015.

[12] G. Nir, I. Farabella, C. Pérez Estrada, C. G. Ebeling, B. J. Beliveau, H. M. Sasaki, S. D. Lee, S. C. Nguyen, R. B. McCole, S. Chattoraj, J. Erceg, J. AlHaj Abed, N. M. C. Martins, H. Q. Nguyen, M. A. Hannan, S. Russell, N. C. Durand, S. S. P. Rao, J. Y. Kishi, P. Soler-Vila, M. Di Pierro, J. N. Onuchic, S. P. Callahan, J. M. Schreiner, J. A. Stuckey, P. Yin, E. L. Aiden, M. A. Marti-Renom, and C. T. Wu. Walking along chromosomes with super-resolution imaging, contact maps, and integrative modeling. *PLoS Genet*, 14(12):e1007872, 12 2018.

[13] S. S. Rao, M. H. Huntley, N. C. Durand, E. K. Stamenova, I. D. Bochkov, J. T. Robinson, A. L. Sanborn, I. Machol, A. D. Omer, E. S. Lander, and E. L. Aiden. A 3D map of the human genome at kilobase resolution reveals principles of chromatin looping. *Cell*, 159(7):1665–1680, Dec 2014.

[14] S. S. P. Rao, S. C. Huang, B. Glenn St Hilaire, J. M. Engreitz, E. M. Perez, K. R. Kieffer-Kwon, A. L. Sanborn, S. E. Johnstone, G. D. Bascom, I. D. Bochkov, X. Huang, M. S. Shamim, J. Shin, D. Turner, Z. Ye, A. D. Omer, J. T. Robinson, T. Schlick, B. E. Bernstein, R. Casellas, E. S. Lander, and E. L. Aiden. Cohesin Loss Eliminates All Loop Domains. *Cell*, 171(2):305–320, Oct 2017.

[15] C. D. Rosencrance, H. N. Ammouri, Q. Yu, T. Ge, E. J. Rendleman, S. A. Marshall, and K. P. Eagen. Chromatin Hyperacetylation Impacts Chromosome Folding by Forming a Nuclear Subcompartment. *Mol Cell*, 78(1):112–126, 04 2020.

[16] S. Sati, B. Bonev, Q. Szabo, D. Jost, P. Bensadoun, F. Serra, V. Loubiere, G. L. Papadopoulos, J. C. Rivera-Mulia, L. Fritsch, P. Bouret, D. Castillo, J. L. Gelpi, M. Orozco, C. Vaillant, F. Pellestor, F. Bantignies, M. A. Marti-Renom, D. M. Gilbert, J. M. Lemaitre, and G. Cavalli. 4D Genome Rewiring during Oncogene-Induced and Replicative Senescence. *Mol Cell*, 78(3):522–538, 05 2020.

[17] G. Stik, E. Vidal, M. Barrero, S. Cuartero, M. Vila-Casadesús, J. Mendieta-Esteban, T. V. Tian, J. Choi, C. Berenguer, A. Abad, B. Borsari, F. le Dily, P. Cramer, M. A. Marti-Renom, R. Stadhouders, and T. Graf. CTCF is dispensable for immune cell transdifferentiation but facilitates an acute inflammatory response. *Nat Genet*, 52(7):655–661, 07 2020.

[18] L. Tian, Y. Shao, S. Nance, J. Dang, B. Xu, X. Ma, Y. Li, B. Ju, L. Dong, S. Newman, X. Zhou, P. Schreiner, E. Tseng, T. Hon, M. Ashby, C. Li, J. Easton, T. A. Gruber, and J. Zhang. Long-read sequencing unveils IGH-DUX4 translocation into the silenced IGH allele in B-cell acute lymphoblastic leukemia. *Nat Commun*, 10(1):2789, 06 2019.

[19] G. Wutz, R. Ladurner, B. G. St Hilaire, R. R. Stocsits, K. Nagasaka, B. Pignard, A. Sanborn, W. Tang, C. Várnai, M. P. Ivanov, S. Schoenfelder, P. van der Lelij, X. Huang,

G. Dürnberger, E. Roitinger, K. Mechtler, I. F. Davidson, P. Fraser, E. Lieberman-Aiden, and J. M. Peters. from WAPL. *Elife*, 9, 02 2020.

[20] Y. Zhang, T. Li, S. Preissl, M. L. Amaral, J. D. Grinstein, E. N. Farah, E. Destici, Y. Qiu, R. Hu, A. Y. Lee, S. Chee, K. Ma, Z. Ye, Q. Zhu, H. Huang, R. Fang, L. Yu, J. C. Izpisua Belmonte, J. Wu, S. M. Evans, N. C. Chi, and B. Ren. Transcriptionally active HERV-H retrotransposons demarcate topologically associating domains in human pluripotent stem cells. *Nat Genet*, 51(9):1380–1388, 09 2019.
